# Correction to: Nrf3 promotes UV-induced keratinocyte apoptosis through suppression of cell adhesion

**DOI:** 10.1038/s41418-019-0437-z

**Published:** 2019-10-22

**Authors:** Beat Siegenthaler, Claudia Defila, Sukalp Muzumdar, Hans-Dietmar Beer, Michael Meyer, Sandra Tanner, Wilhelm Bloch, Volker Blank, Matthias Schäfer, Sabine Werner

**Affiliations:** 1 Department of Biology, Institute of Molecular Health Sciences, ETH Zurich, Zurich, 8093 Switzerland; 2 Department of Dermatology, University Hospital Zurich, Zurich, CH-8091 Switzerland; 3 Department of Molecular and Cellular Sport Medicine, German Sport University Cologne, Cologne, 50933 Germany; 4 Lady Davis Institute for Medical Research, McGill University, Montreal, Canada

**Keywords:** Cell adhesion, Experimental models of disease

**Correction to: Cell Death & Differentiation**

10.1038/s41418-018-0074-y

The original PDF version of this article incorrectly showed the copyright holder to be ‘ADMC Associazione Differenziamento e Morte Cellulare 2018’, when the correct copyright holder is ‘The Authors 2018’. This has been corrected in the PDF version of the article. The HTML version was correct from the time of publication.

